# Supplementary material for: MAPK Inhibition Requires Active RAC1 Signaling to Effectively Improve Iodide Uptake by Thyroid Follicular Cells
Source: Cancers (Basel). 2021 Nov 22;13(22):5861. doi: 10.3390/cancers13225861 (PMC8616057; doi:10.3390/cancers13225861)
Supplement: Supplementary file 1 [file cancers-13-05861-s001.zip › cancers-1477593-supplementary.pdf]

cancers

MDPI

Supplementary Materials: MAPK Inhibition Requires Active RAC1 Signaling to Effectively Improve Iodide Uptake by Thyroid Follicular Cells

Márcia Faria, Rita Domingues, Maria João Bugalho, Paulo Matos, Ana Luísa Silva

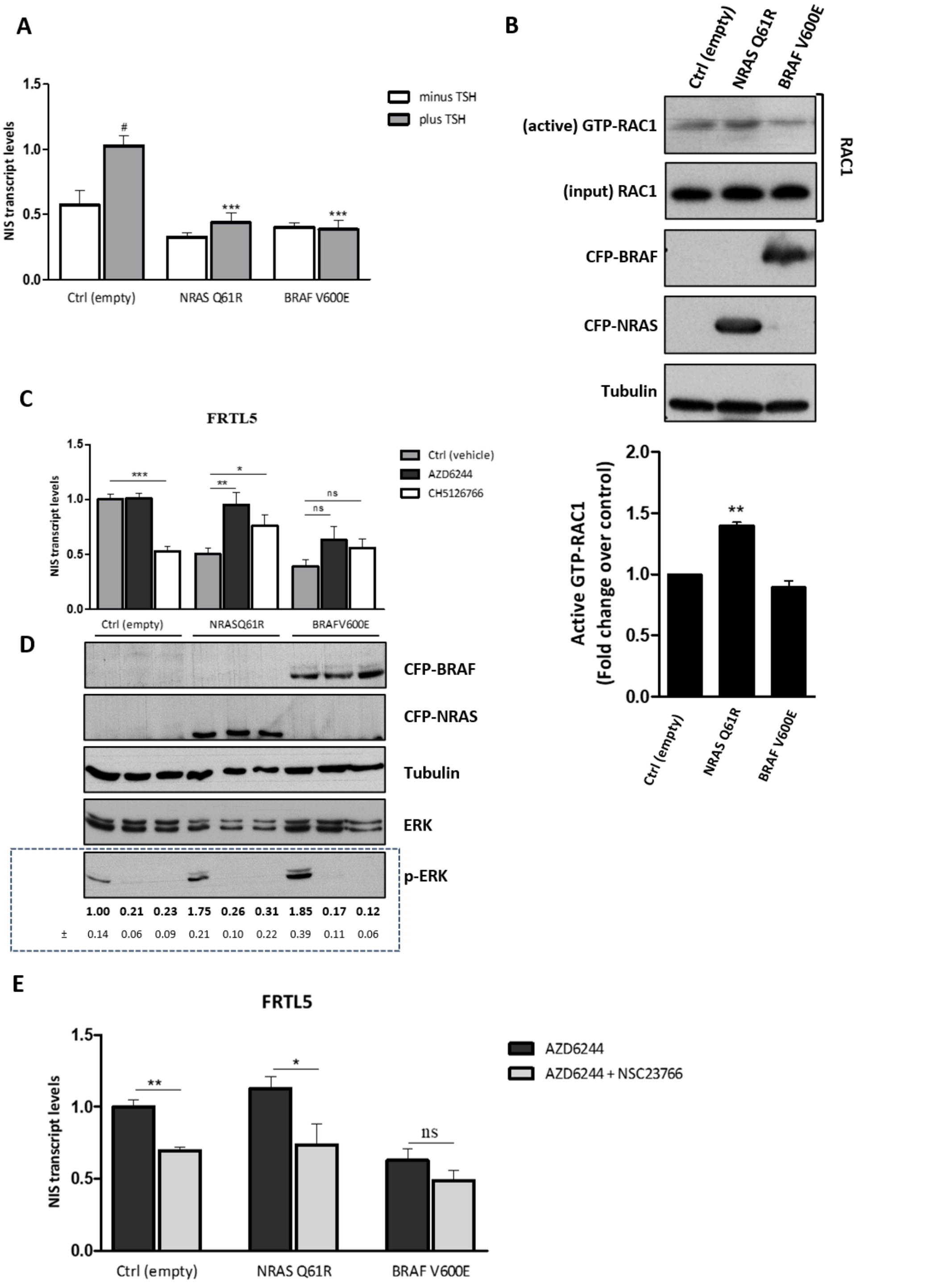

Figure S1. Effect of NRAS and BRAF mutants, MEK1/2 inhibition and RAC signaling on NIS transcriptional expression in the non-neoplastic, TSH-responsive thyroid follicular FRTL5 cell line. (**A**)

FRTL5 cells were transduced with empty, NRAS-Q61R or BRAF-V600E constructs and subjected to a 24 h starvation period (minus TSH) followed by stimulation with TSH (1 mU/mL for 48 h; plus TSH). NIS mRNA levels were quantified by RT-qPCR plotted as fold differences relative to mock transfected [Ctrl (empty)] cells, treated with TSH. Values are the mean ± SEM of five independent assays. One-way ANOVA analysis detected significant differences between the different conditions ($F = 11.11$; $p < 0.0001$). Post-hoc Tukey's tests were used to identify significant variations from Ctrl (empty) conditions (# $p \leq 0.05$ relative to minus TSH; *** $p \leq 0.001$ relative to plus TSH). (**B**) Endogenous RAC1 activation status in Ctrl (empty)-, NRAS-Q61R- and BRAF-V600E-expressing cells was assessed by monitoring the active, GTP-bound fraction of RAC1 by CRIB domain pull-down assay. Total (input) and active RAC1 levels were assessed by western blot using anti-RAC1 primary antibody (quantified in the plot to the right; $F = 69.14$, $p = 0.022$; ** $p \leq 0.01$ relative to ctrl [empty]). NRAS and BRAF expression was detected using anti-GFP primary antibody. Alpha-tubulin was used as loading control. (**C**) FRTL5 transduced cells were serum-starved for 24 h followed by stimulation with TSH (1 mU/mL for 48 h), in the presence or absence of either AZD6244 (10 μM for 48 h) or CH5126766 (10 μM for 48 h). NIS mRNA levels were quantified by RT-qPCR and plotted as in Figure 2. Comparisons were made using a two-tailed Student's t-test between AZD6244-treated or CH5126766-treated versus untreated setting (ns—not significant; * $p \leq 0.05$; ** $p \leq 0.01$; *** $p \leq 0.001$). (**D**) NRAS and BRAF expression was assessed by western blot using anti-GFP primary antibody. MAPK activation status was assessed by monitoring phosphorylated ERK1/2 vs. total ERK1/2 levels by Western blot [quantified below the respective panel (means ± SEM of three independent assays)], using anti-phospho-ERK1/2 and anti-ERK1/2 primary antibodies, respectively. Tubulin was used as loading control. (**E**) Transduced FRTL5 cells were serum-starved for 24h, stimulated with TSH (1 mU/mL for 48 h) and treated with AZD6244 (10 μM for 48 h), in the presence or absence of RAC1 inhibitor (NSC23766; 100 μM for 24 h). NIS mRNA levels were quantified by RT-qPCR and plotted as in Figure 3. Comparisons were made using a two-tailed Student's t-test between NSC23766-treated versus untreated setting (ns—not significant; * $p \leq 0.05$; ** $p \leq 0.01$).

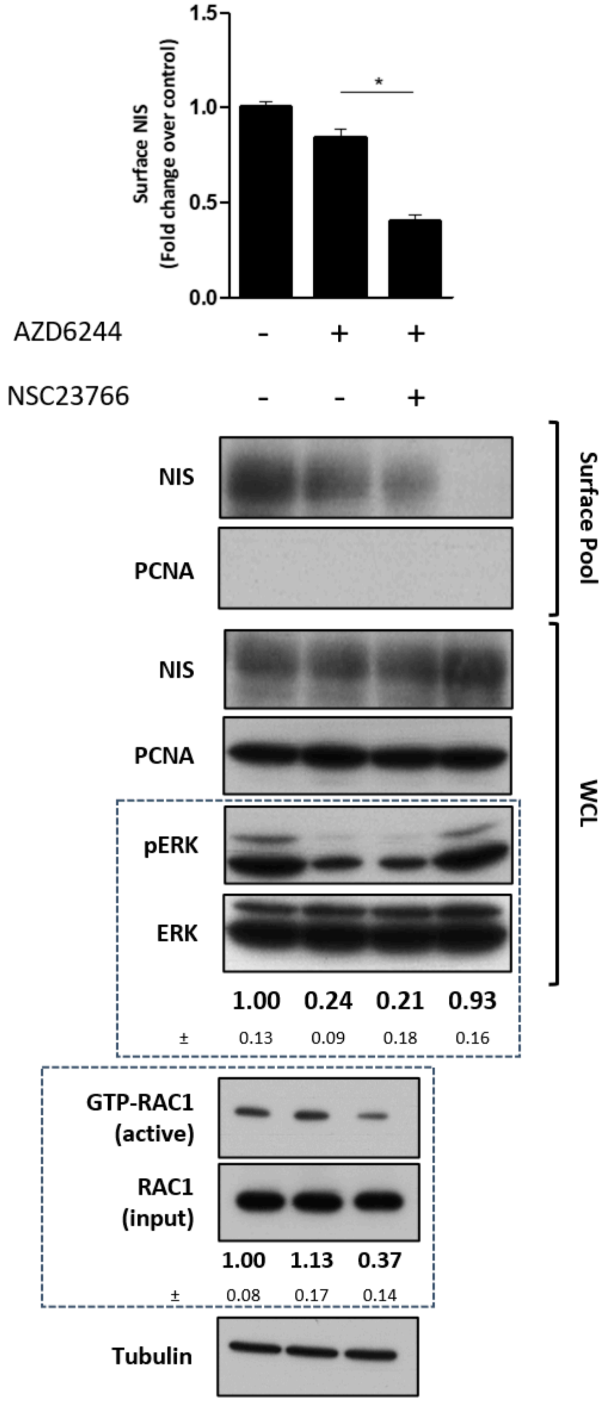

Figure S2. Impact of MEK and RAC1 inhibition on endogenous NIS levels at the plasma membrane of PCCL3 cells. PCCL3 cells were subjected to a 24h starvation period and then treated with TSH for 48 h, in the presence or absence of AZD6244 (10μM for 1h), alone or in combination with NSC23766 (100 μM for 1h). The activation status of endogenous RAC1 was monitored using a CRIB domain pull-down assay. Total (input) and active RAC1 levels were assessed using anti-RAC1 primary antibody [quantified below the respective panel (means ± SEM of three independent assays)]. Tubulin was used as loading control. MAPK activation status was assessed by monitoring phosphorylated ERK1/2 vs. total ERK1/2 levels by Western blot [quantified below the respective panel (means ± SEM of three independent assays)], using anti-phospho-ERK1/2 and anti-ERK1/2 primary antibodies, respectively. Cell surface proteins were biotinylated and both whole-cell lysates (WCL) and surface protein fraction were analyzed by western blot. 'no Biotin' condition, corresponding to cells that were not incubated with biotin, was used as control for the capture of non-biotinylated proteins. Total and surface NIS protein levels were detected using anti-NIS primary antibody. PCNA expression served as loading (WCL) and negative control (Surface pool). Plotted values are means ± SEM of three independent assays. Comparisons between AZD6244-alone or AZD6244+NSC23766-treated cells were made using a two-tailed Student's t-test (* $p \leq 0.05$). In all panels, WB bands were quantified by densitometry analysis using ImageJ software.

kDa
180
135
100
72
60
45
35
25
20
15
10

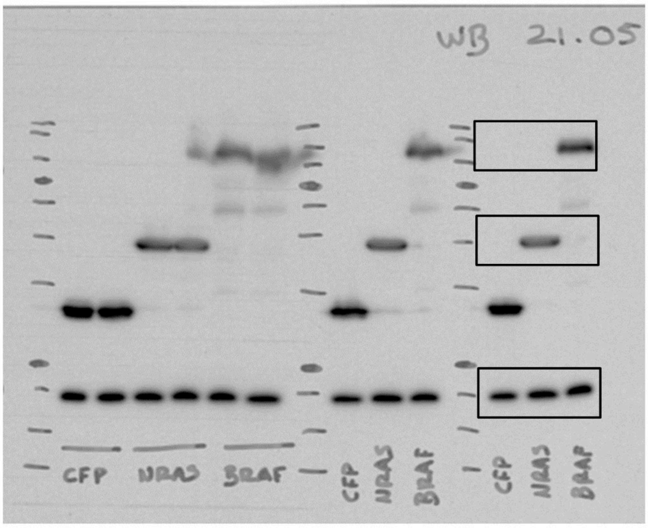

CFP-BRAF

CFP-NRAS

(input) RAC1

kDa
180
135
100
72
60
45
35

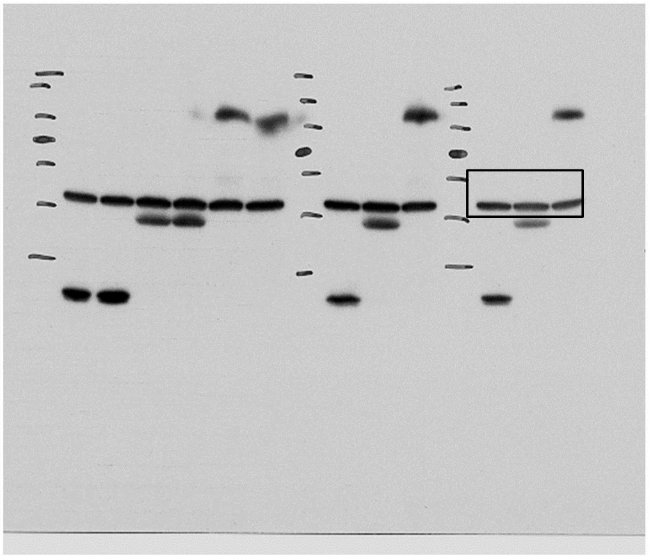

Tubulin
(Reprobed Membrane)

kDa
35
25
20

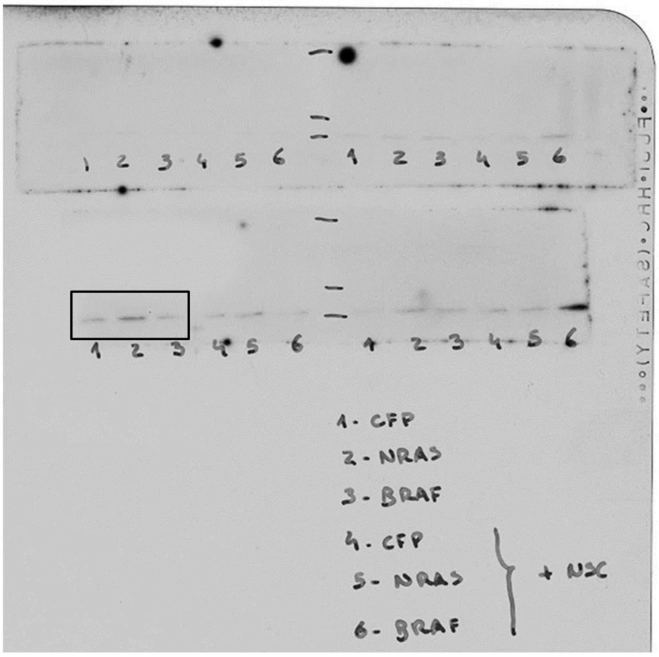

(pull-down) GTP-RAC1

Uncropped WB original images for Figure 1

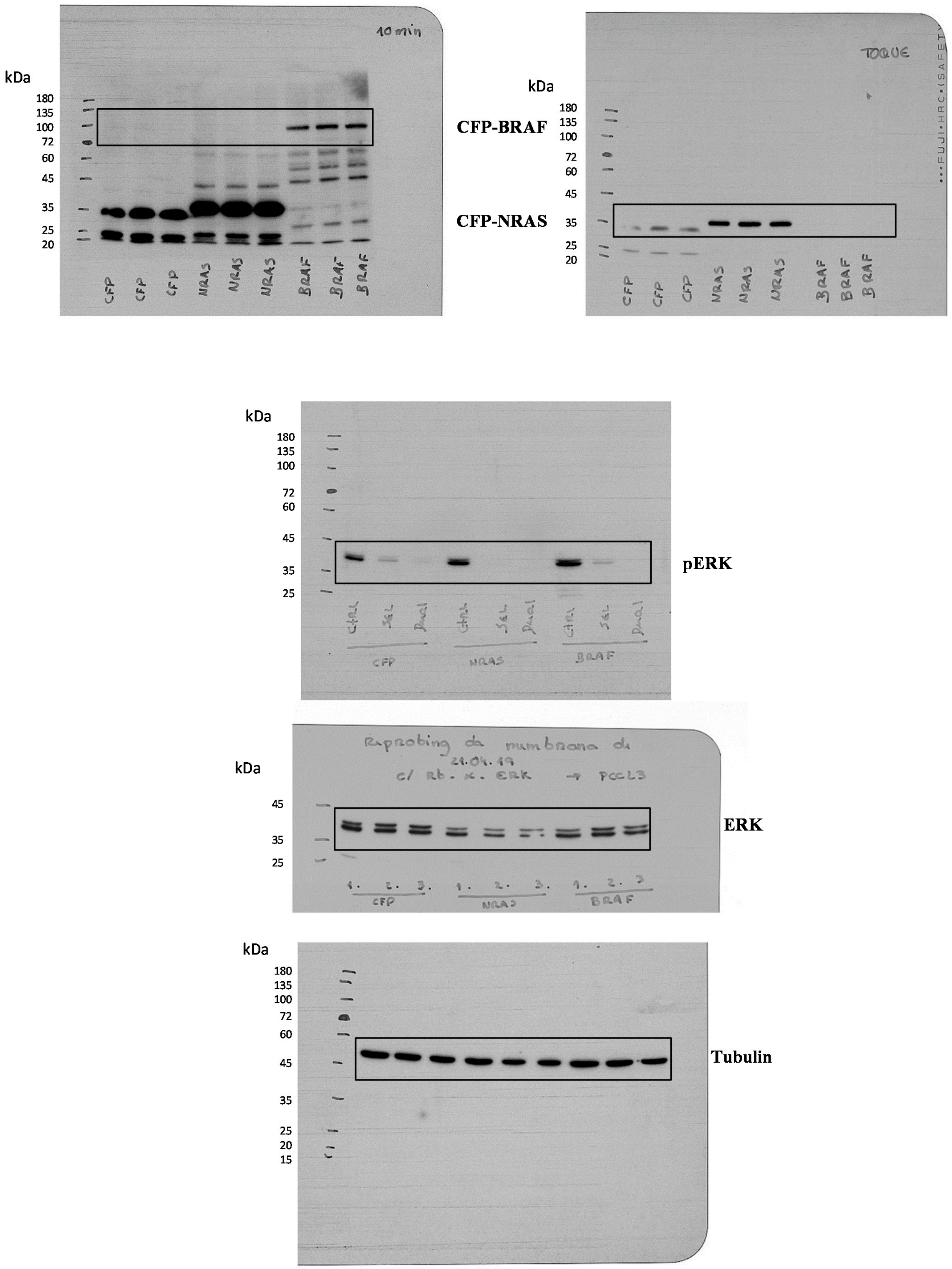

Uncropped WB original images for Figure 2

A

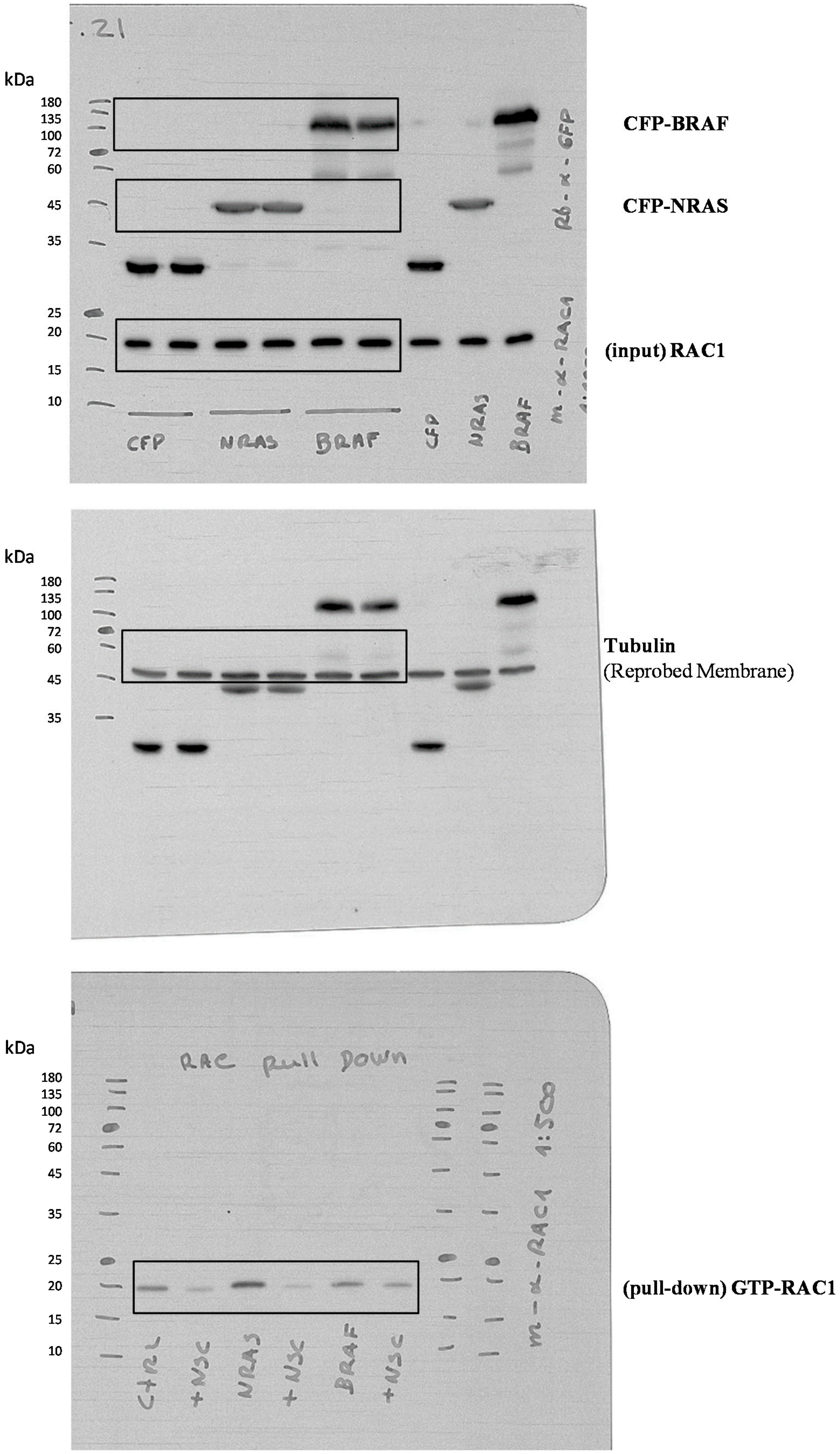

Uncropped WB original images for Figure 3

C

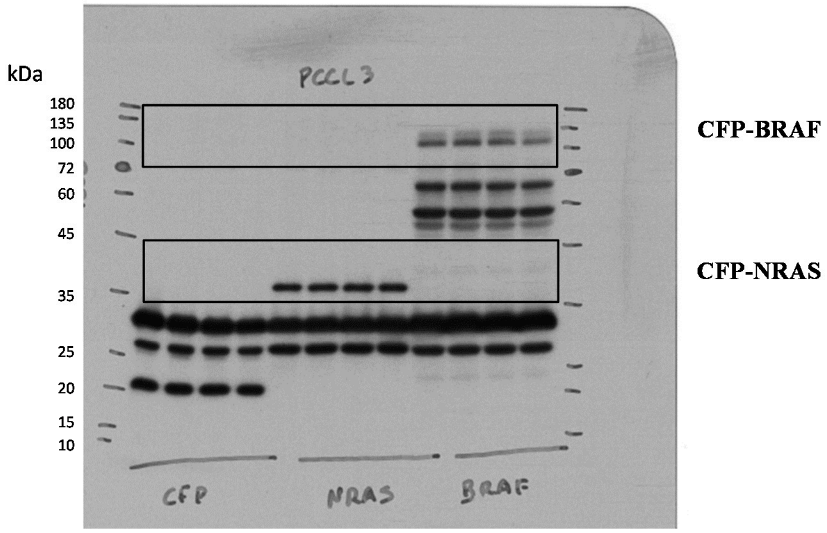

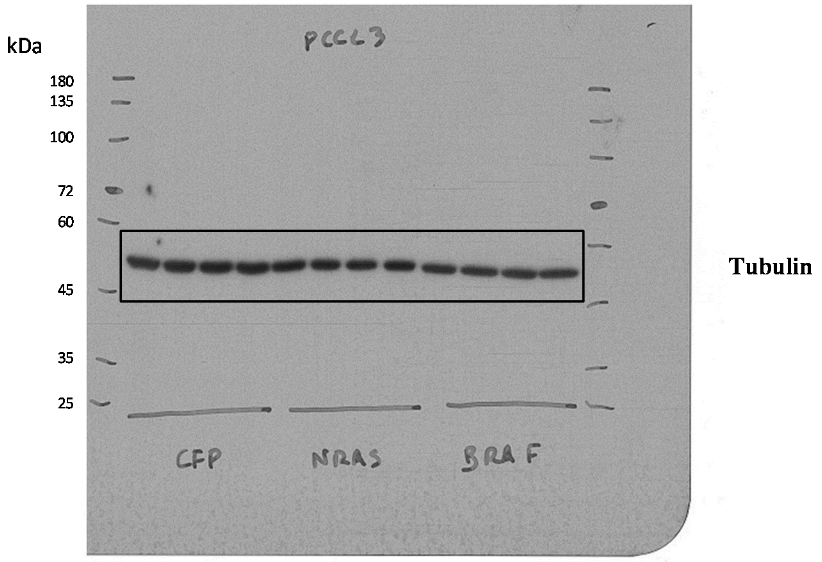

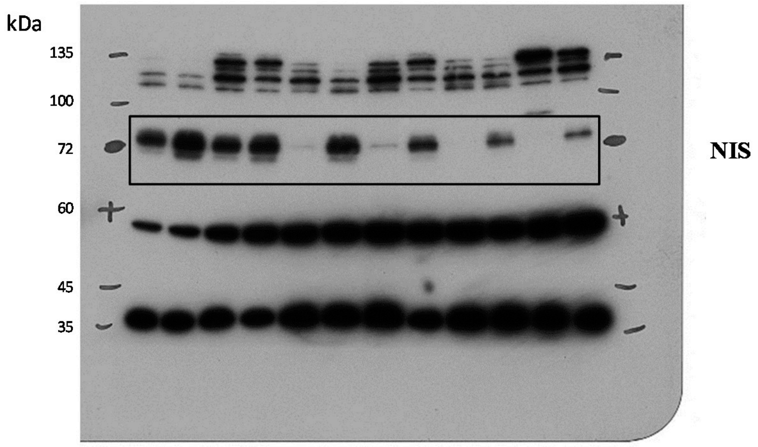

Uncropped WB original images for Figure 3

GFP - RAC1

Endogenous RAC1

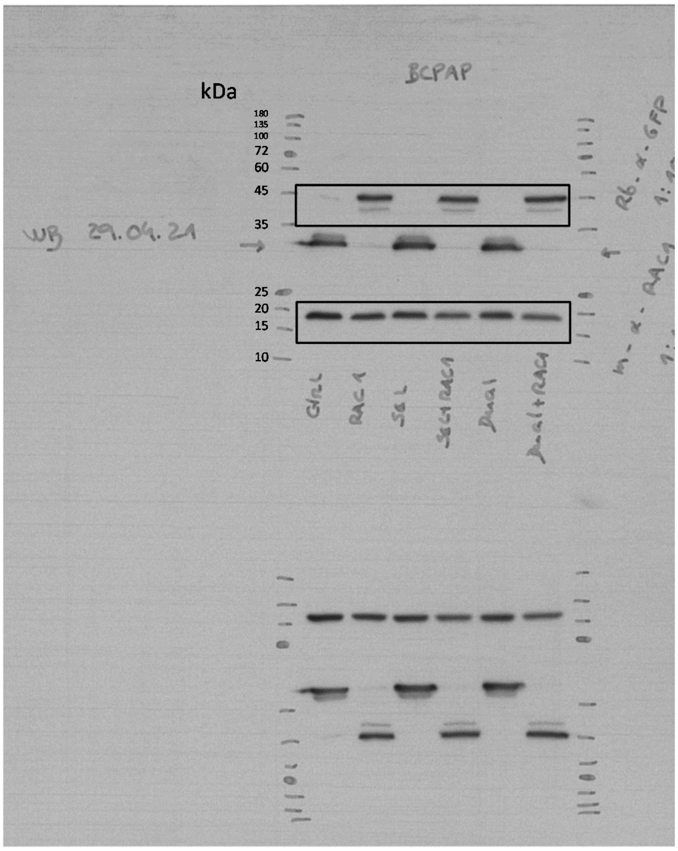

kDa
180
135
100
72
60
45
35
25
20

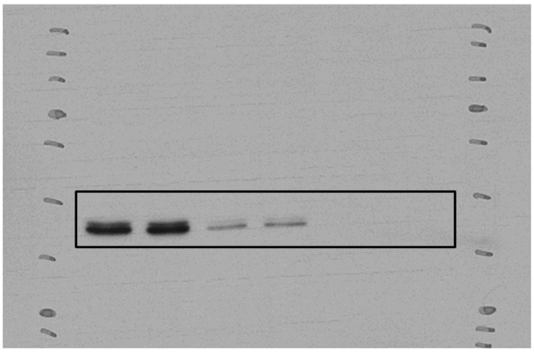

pERK

kDa
45
35
25
20
15

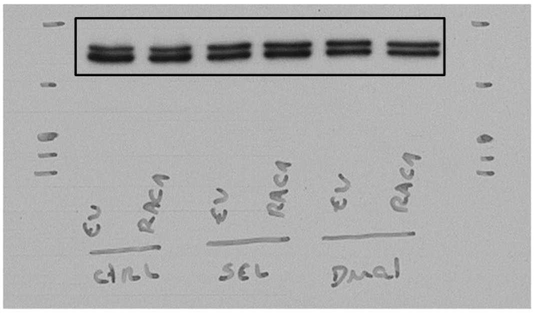

ERK

kDa
180
135
100
72
60
45
35

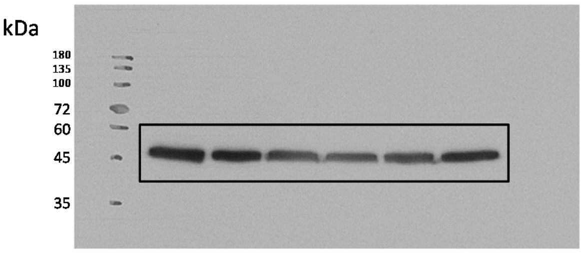

Tubulin

Uncropped WB original images for Figure 4

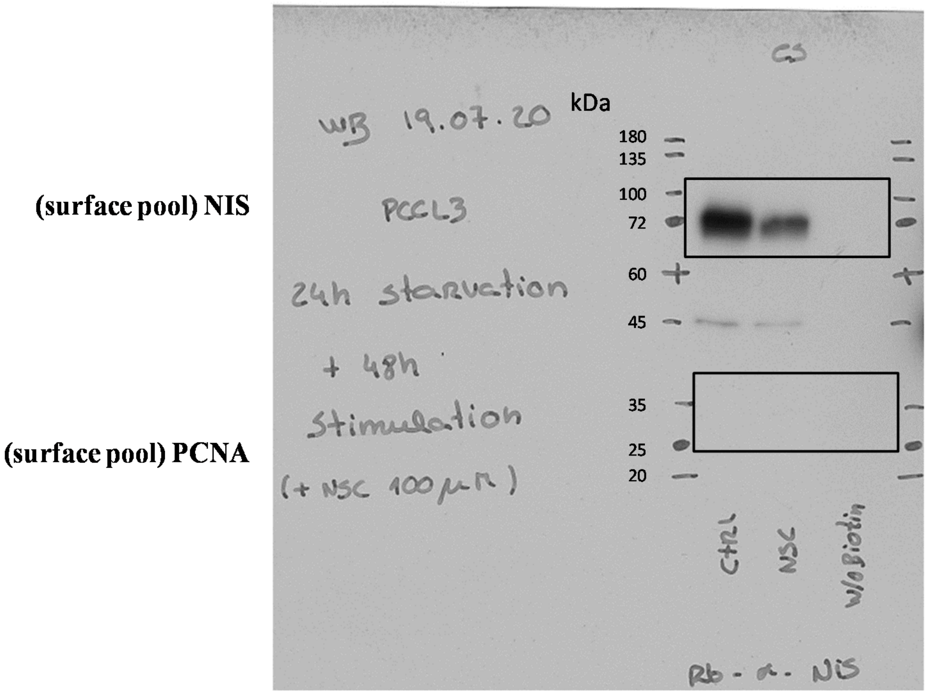

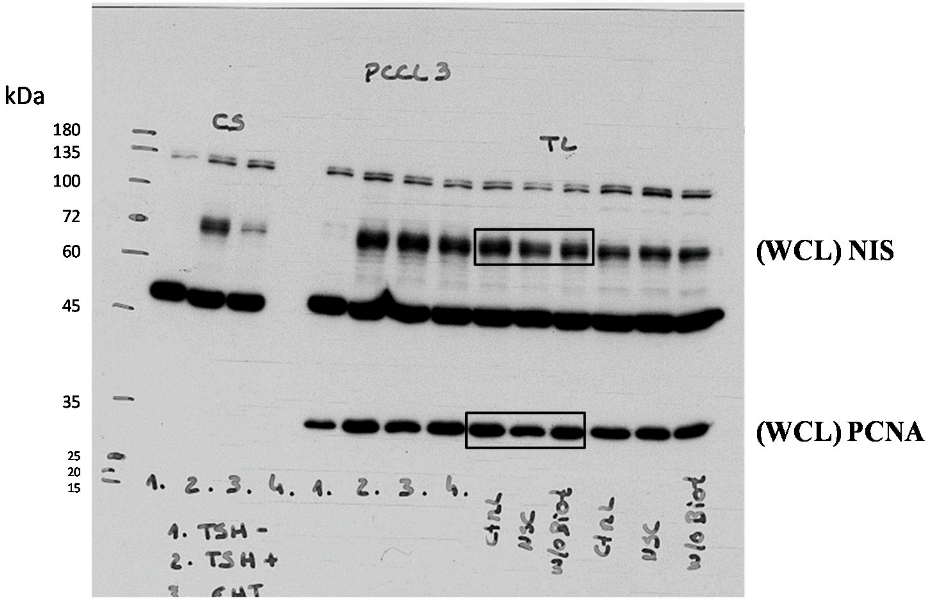

Uncropped WB original images for Figure 5

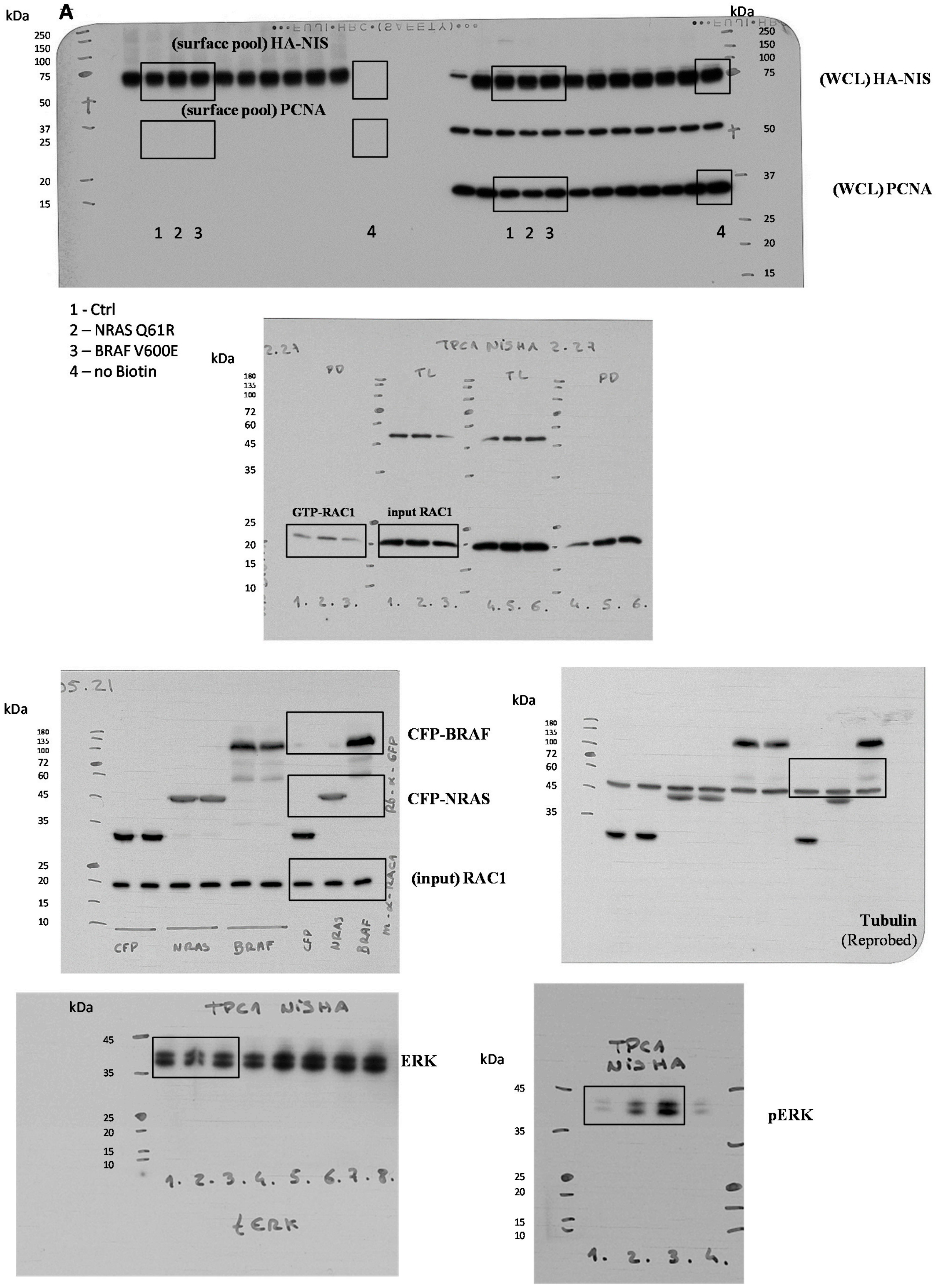

Uncropped WB original images for Figure 6

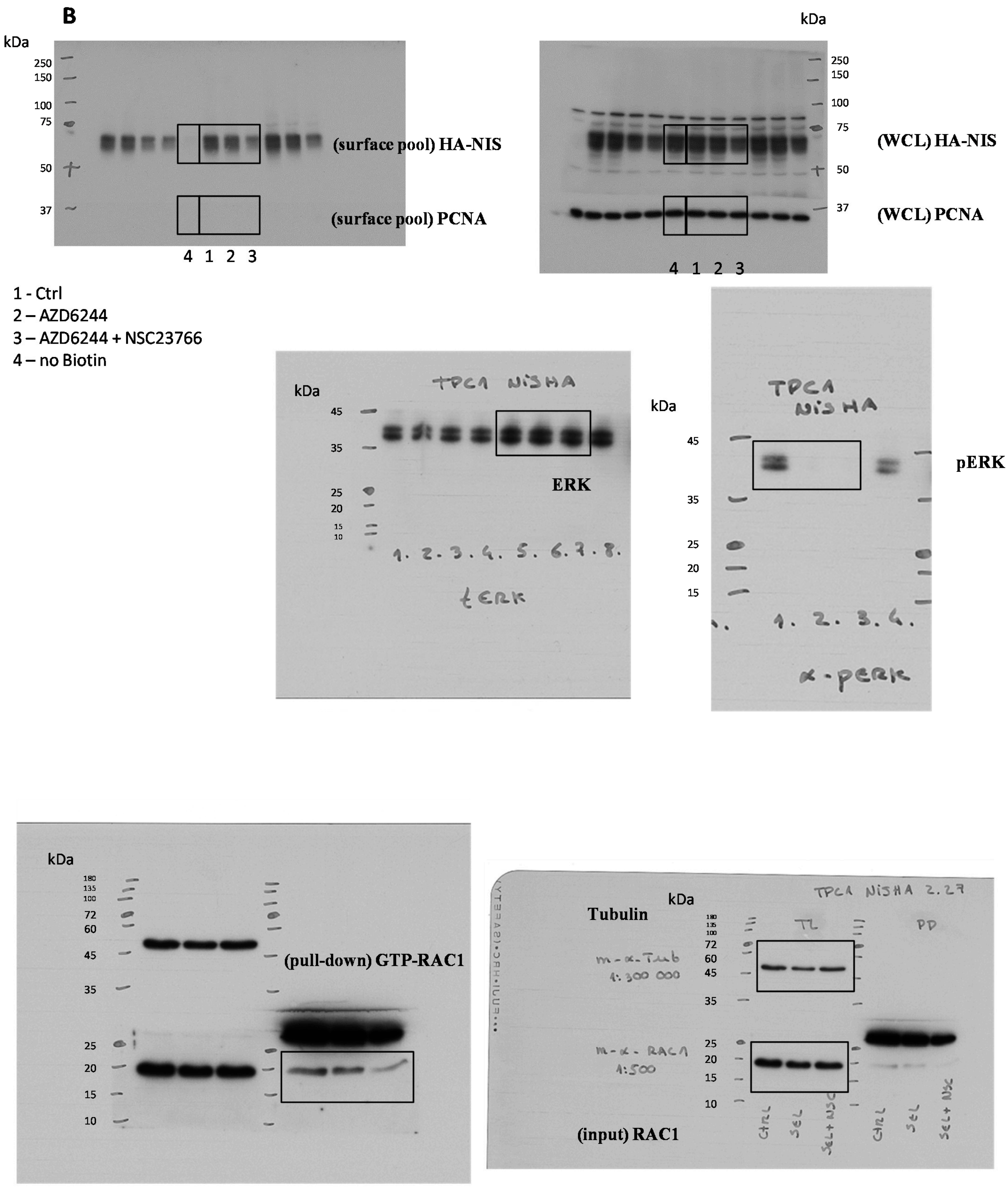

Uncropped WB original images for Figure 6

B

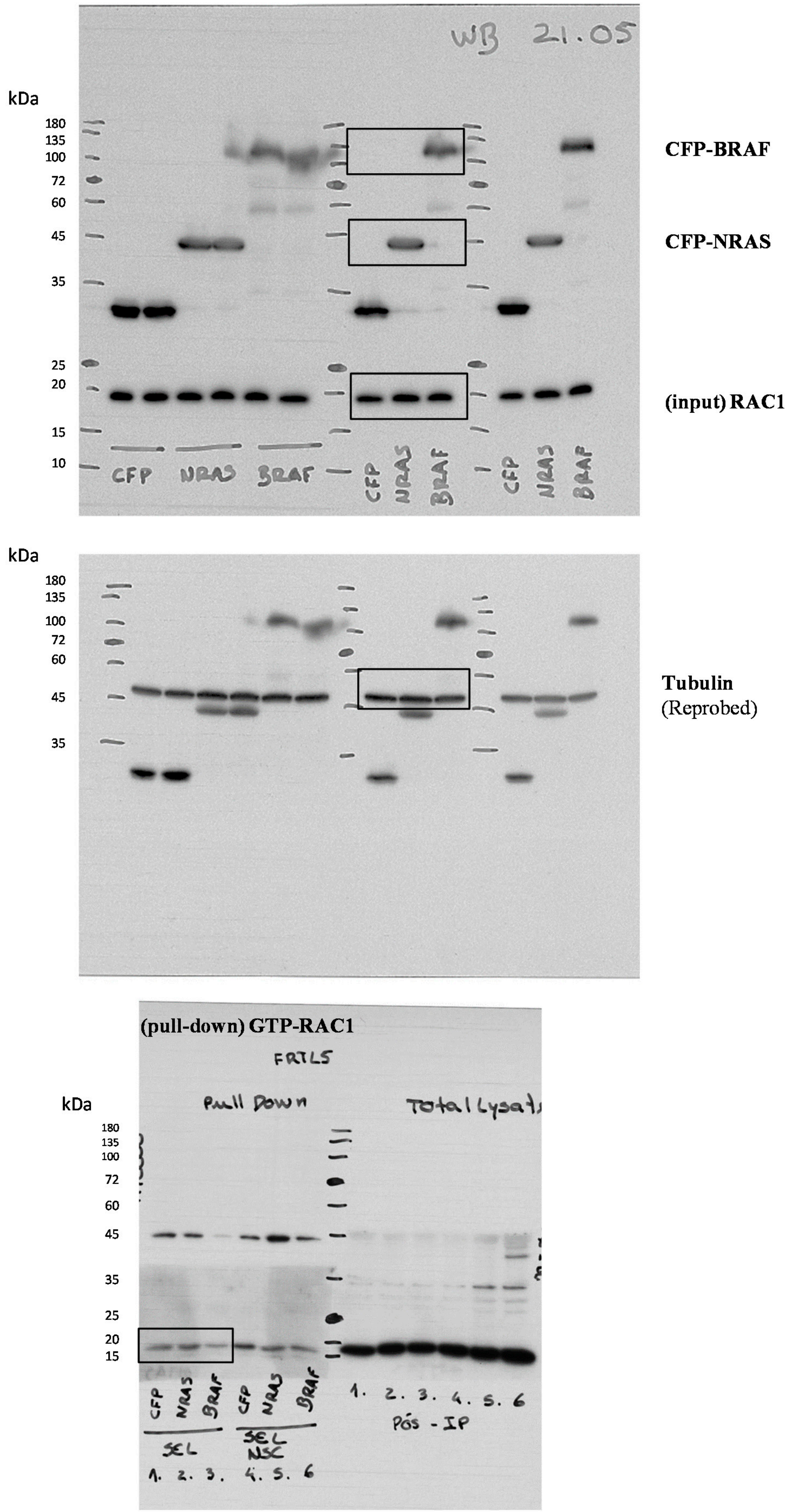

Uncropped WB original images for Figure S1

D

CFP-BRAF | **CFP-NRAS**

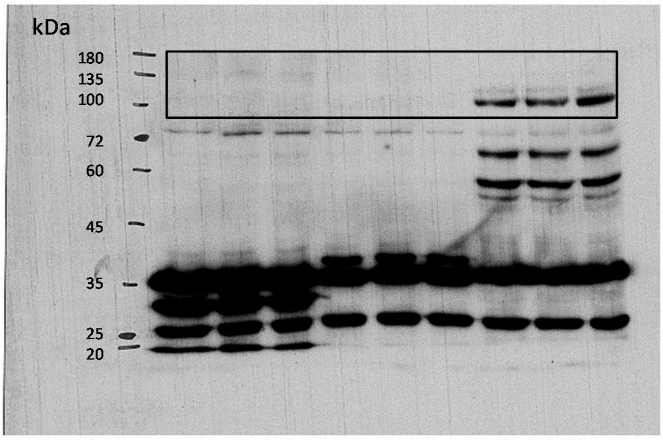

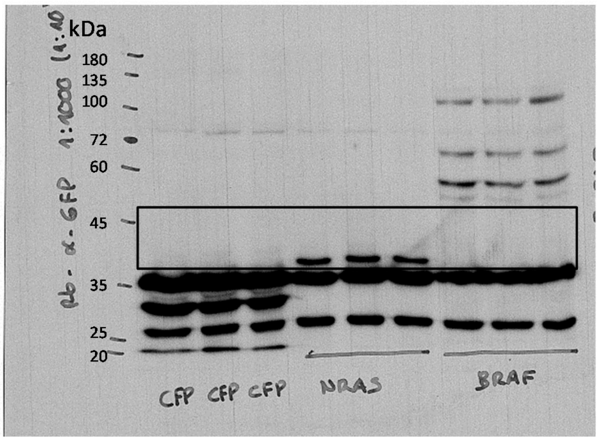

pERK

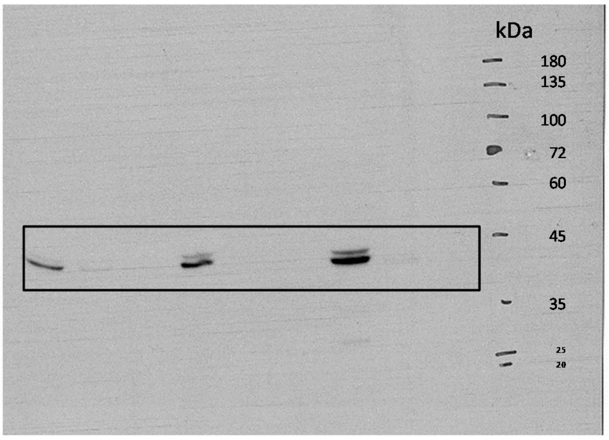

tERK

kDa

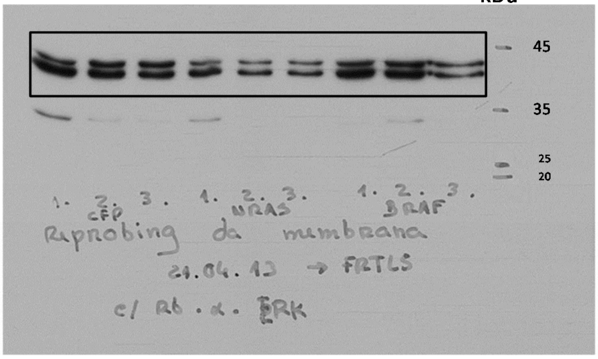

Tubulin

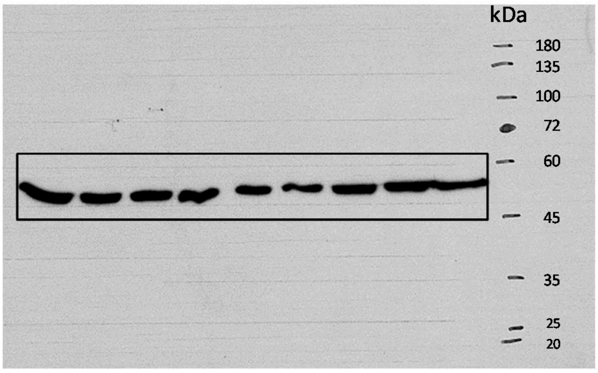

Uncropped WB original images for Figure S1

PCCL3

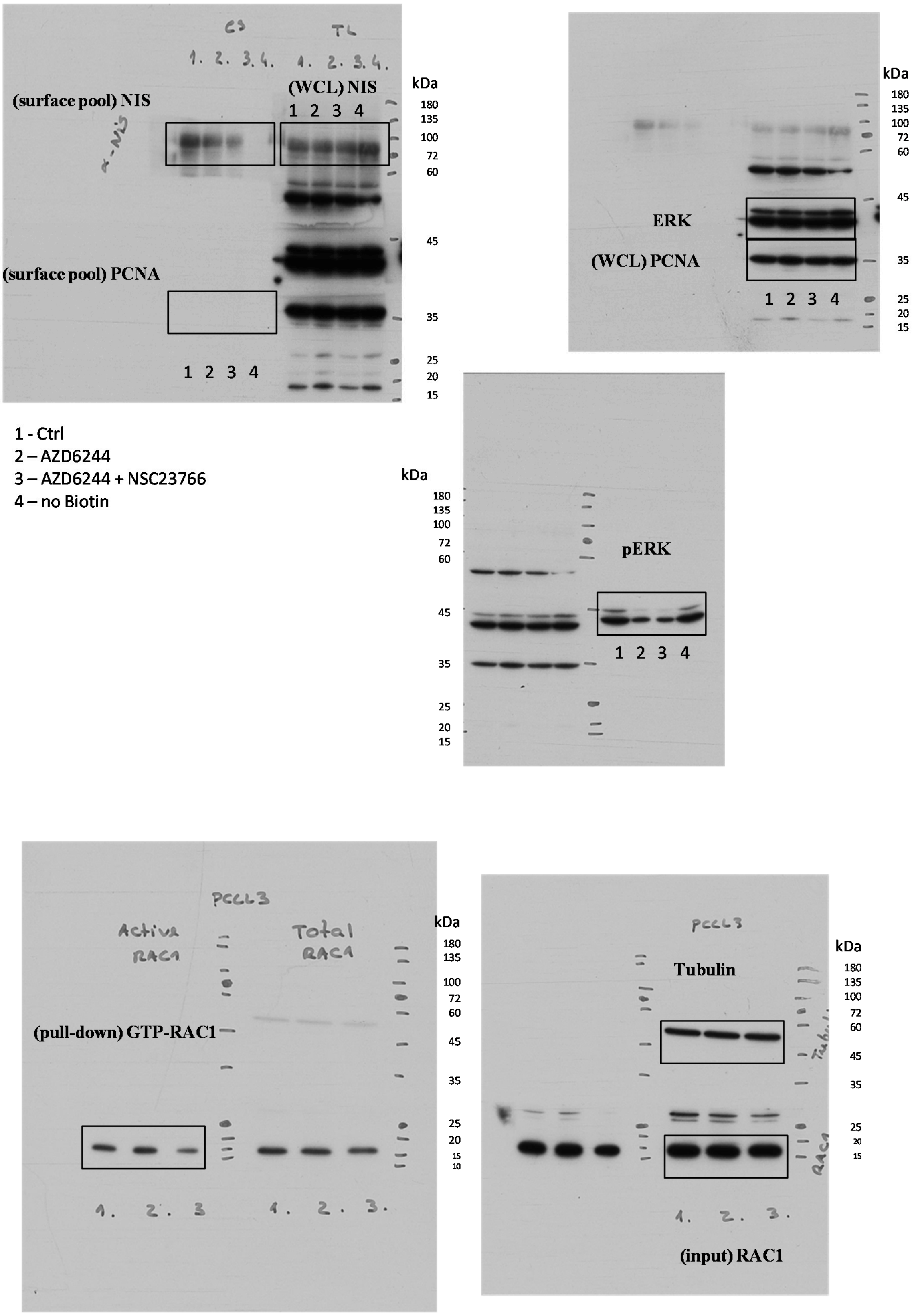

Uncropped WB original images for Figure S2

Figure S3. Uncropped WB original images.
